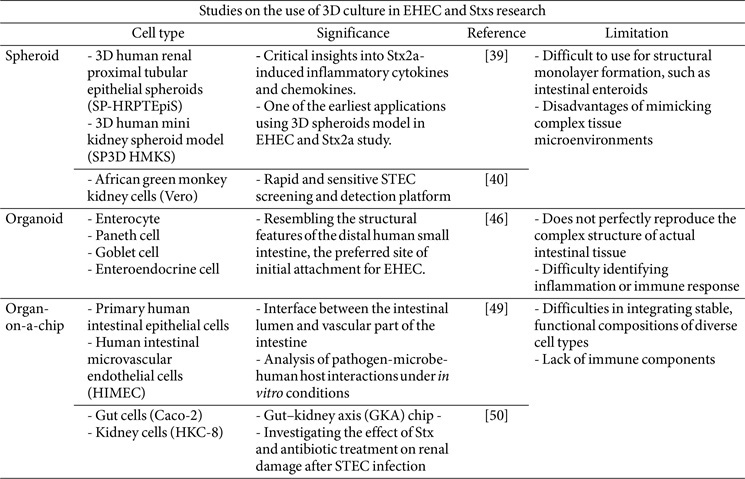# Advanced Three-Dimensional Cell Culture Models for Investigating Enterohaemorrhagic *Escherichia coli* Pathogenesis and Shiga Toxin-Mediated Injury

**DOI:** 10.4014/jmb.2507.07021

**Published:** 2025-09-05

**Authors:** So-Hyeon Park, Mirim Kim, Yu-Jin Jeong, Moo-Seung Lee

**Affiliations:** 1 Environmental Diseases Research Center, Korea Research Institute of Bioscience and Biotechnology, Daejeon 34141, Republic of Korea; 2 Department of Biochemistry, Chungnam National University, Daejeon 34134, Republic of Korea; 3 Department of Biomolecular Science, KRIBB School of Bioscience, Korea University of Science and Technology (UST), Daejeon 34113, Republic of Korea

**Keywords:** EHEC, STEC, Shiga toxin, 3D cell culture systems, spheroids, organoids, organ on a chip

## Abstract

Enterohemorrhagic *Escherichia coli* (EHEC), a pathotype within the Shiga toxin-producing *E. coli* (STEC) group, is a major etiological agent of severe gastrointestinal illness and life-threatening sequelae, including hemolytic uremic syndrome. Although insights into EHEC pathogenesis have been gained through traditional 2D cell culture systems and animal models, these platforms are limited in their ability to recapitulate human-specific physiological responses and tissue-level interactions. Recent progress in three-dimensional (3D) cell culture systems, such as spheroids, organoids, and organ-on-a-chip (OoC) technologies, has enabled more physiologically relevant *in vitro* models for investigating host-pathogen dynamics. These advanced platforms offer improved modeling of human tissue architecture, cellular heterogeneity, and microenvironmental complexity, thereby providing novel perspectives on Shiga toxin-mediated damage in intestinal and renal systems. This review synthesizes current applications of 3D culture systems in EHEC research, critically evaluates their advantages and limitations, and outlines future directions for enhancing mechanistic understanding and translational relevance in STEC-associated disease modeling.

## Pathogenic *Escherichia coli*

*Escherichia coli* is a facultative anaerobic Gram-negative bacterium that typically resides as a commensal in the human gastrointestinal tract. While most *E. coli* strains contribute to intestinal homeostasis, certain lineages acquire virulence genes via plasmids, pathogenicity islands, or bacteriophages, enabling them to cause a broad range of human diseases. These strains, known as pathogenic *E. coli*, are broadly divided into extraintestinal pathogenic *E. coli* (ExPEC) and diarrheagenic *E. coli* (DEC) based on the site and nature of infection.

Within DEC, at least seven major pathotypes have been identified, each exhibiting distinct mechanisms of host colonization and disease progression [1]:

**1. Enteropathogenic *E. coli* (EPEC):**

A leading cause of infantile diarrhea in developing countries, EPEC induces attaching-and-effacing (A/E) lesions in the small intestine via a type III secretion system and the intimin–Tir interaction. Unlike other pathotypes, it does not produce Shiga toxins or enterotoxins.

**2. Enterotoxigenic *E. coli* (ETEC):**

Responsible for traveler’s diarrhea and a major cause of childhood diarrhea, ETEC adheres to the intestinal mucosa and secretes heat-labile (LT) and/or heat-stable (ST) enterotoxins, resulting in watery, secretory diarrhea.

**3. Enterohemorrhagic *E. coli* (EHEC):**

A highly virulent pathotype, most notably serotype O157:H7, EHEC produces potent Shiga toxins (Stx1 and Stx2) and also forms A/E lesions. These mechanisms can lead to hemorrhagic colitis and the life-threatening complication hemolytic uremic syndrome (HUS).

**4. Enteroaggregative *E. coli* (EAEC):**

Characterized by a “stacked-brick” adherence pattern, EAEC promotes persistent diarrhea through aggregative adherence fimbriae (AAF), biofilm formation, and induction of pro-inflammatory host responses.

**5. Enteroinvasive *E. coli* (EIEC):**

Genetically similar to Shigella, EIEC invades colonic epithelial cells and causes a dysentery-like illness marked by fever, abdominal cramps, and bloody diarrhea.

**6. Diffuse-Adhering *E. coli* (DAEC):**

Associated primarily with diarrhea in children, DAEC exhibits a diffuse adherence pattern to intestinal epithelial cells. Its precise pathogenic mechanisms remain incompletely defined.

**7. Adherent-Invasive *E. coli* (AIEC):**

An emerging pathotype implicated in Crohn’s disease, AIEC invades intestinal epithelial cells and replicates within macrophages without inducing host cell death, thereby contributing to chronic intestinal inflammation. These categories are further distinguished by their O (somatic), H (flagellar), and K (capsular) antigens, which serve as epidemiological markers. The vast genetic plasticity of *E. coli* facilitates ongoing recombination and the emergence of hybrid or hypervirulent strains that defy classical categorization. Among these, EHEC stands out for its ability to cause severe systemic disease, largely attributed to the elaboration of Shiga toxins, placing it at the center of this review.

## The Clinical and Molecular Significance of the STEC and Shiga Toxin Study

Shiga toxin-producing *E. coli* (STEC) are distinguished by their ability to secrete Shiga toxins, which are implicated in serious clinical outcomes such as hemorrhagic colitis and HUS—a leading cause of acute kidney injury in children [2]. In addition to renal damage, HUS can lead to neurological complications such as seizures, stroke, and altered mental status, as well as systemic manifestations including hypertension, pancreatitis, and cardiovascular dysfunction [3]. The *stx* gene, originally identified in *Shigella dysenteriae* serotype 1, shares genetic and structural homology with toxins produced by EHEC strains [4]. Upon infection, Shiga toxin damages intestinal epithelial cells (IECs), crosses the epithelial barrier, and enters systemic circulation, where it binds to globotriaosylceramide (Gb3) [5, 6]. Gb3 is expressed not only on intestinal and renal epithelial cells but also on certain immune cells, including monocytes, macrophages, and B cells, which may serve as carriers of the toxin [7-9]. Its high abundance in the intestine, kidney, and brain explains why these organs are principal targets of Shiga toxin–mediated damage [10, 11]. Following receptor binding, Stx is internalized by endocytosis and triggers a cascade of host cellular responses, including unfolded protein response, endoplasmic reticulum (ER) stress, ribotoxic stress, and inflammatory signaling, ultimately leading to apoptosis and pyroptosis [12, 13].

According to the WHO, foodborne STEC infections caused more than one million illnesses and approximately 13,000 disability-adjusted life years (DALYs) globally in 2020 [8]. The majority of outbreaks occurred in the Americas (78%) and Europe (18%), with a smaller proportion in the Western Pacific (4%) [9]. Although more than 400 STEC serotypes have been identified, EHEC O157:H7 remains the most common and virulent, having repeatedly caused large-scale foodborne outbreaks since the 1980s [10-12]. However, the 2011 outbreak in Germany, driven by the hybrid EHEC O104:H4 strain, highlighted critical gaps in surveillance and diagnostics. This strain combined enteroaggregative features (such as AAF/I fimbriae, strong adherence to HEp-2 cells, and biofilm formation) with Shiga toxin production, making it undetectable by conventional diagnostic methods [14, 15]. This event, which led to thousands of cases and dozens of fatalities, underscored the emergence of novel hybrid variants and the urgent need for improved diagnostic tools and research into the diversity of EHEC pathogenesis.[Fig F1]

## The Need for Advanced *in vitro* Models in EHEC Research

Despite their contributions, conventional 2D monolayers and animal models exhibit major limitations in mimicking human physiology and disease mechanisms. Standard 2D cultures lack the cellular complexity and 3D tissue architecture necessary for recapitulating host-pathogen interactions. Similarly, murine models do not fully represent human disease due to differences in receptor expression, notably the limited expression of Gb3 in mouse glomeruli [16].

DesRochers, Teresa M *et al*., demonstrated that kidney-derived 3D tissue models exposed to Stx2 exhibited significantly elevated expression of kidney injury markers, including KIM-1 and IL-8, compared to 2D models—despite comparable levels of cytotoxicity [17]. Although non-human primates offer greater physiological relevance, their use is limited due to high cost, ethical concerns, and the need for specialized facilities [10]. Given these limitations, 3D cell culture platforms such as spheroids, organoids, and organ-on-a-chip systems are emerging as powerful tools to study STEC infections in human-relevant contexts.

These models more accurately simulate tissue microenvironments, enabling better mechanistic insights and translational relevance in studies of Stx pathogenesis.

## Overview of 3D Host Cell Models

Conventional *in vitro* and *in vivo* model systems, including immortalized cell lines and animal models, have historically provided foundational insights in biomedical research areas such as cellular signaling, therapeutic target identification, and drug development for both cancer and infectious diseases [18]. However, accumulating evidence has revealed species-specific differences, particularly in areas like neural development, metabolism, and pharmacological response, which are not adequately replicated in non-human models. In response, 3D *in vitro* models have garnered growing interest as more physiologically relevant platforms capable of bridging these translational gaps. In this section, we focus on three representative 3D culture platforms—spheroids, organoids, and organ-on-a-chip (OoC) systems—that have been directly applied to EHEC and Shiga toxin research.

Spheroids are self-assembled, three-dimensional aggregates of various cell types that form under non-adherent culture conditions. These models promote enhanced cell–cell and cell–extracellular matrix (ECM) interactions through upregulation of adhesion molecules such as E-cadherin and integrin-mediated ECM binding [19, 20]. Multiple techniques are available for generating spheroids, including pellet culture, hanging drop, liquid overlay, spinner flasks, rotating-wall vessels (RWVs), magnetic levitation, and microfluidics. Spheroids can be broadly classified into scaffold-free and scaffold-assisted types depending on the method of assembly [21, 22]. Scaffold-free techniques offer simplicity and cost-effectiveness, whereas scaffold-based systems are more commonly used in tissue engineering applications due to their ability to mimic ECM structure [19]. Unlike 2D cultures, spheroids exhibit improved spatial organization and better recapitulate native tissue characteristics, enabling more accurate modeling of *in vivo* cell signaling and secretory activity [20-22].[Fig F2]

Organoids are generated from induced pluripotent stem cells (iPSCs) or adult stem cells (ASCs) by mimicking developmental or regenerative processes that recapitulate key architectural and functional features of their tissue of origin [18, 23]. iPSCs are reprogrammed somatic cells that can differentiate into various tissue types, including the brain, retina, stomach, lungs, and intestines under the influence of ECM and niche-specific growth factors [24, 25]. One of their key advantages is the ability to generate organoids without needing primary tissue, which is often limited by logistical and ethical constraints. However, iPSC-derived organoids often resemble fetal tissue and may lack features of fully mature epithelium or region-specific cellular identity [26]. In contrast, ASC-derived organoids are established directly from patient tissues or biopsies and retain greater fidelity to adult stem cell niche environments. These models more accurately reflect *in vivo* epithelial diversity and have been used to model disease-specific tissue responses, especially in gastrointestinal research [18, 25]. The landmark study by Hans Clevers' group in 2009 demonstrated that Lgr5+ stem cells isolated from intestinal crypts could be cultured long-term using Matrigel and growth factor-enriched media, leading to the development of functional intestinal organoids [27]. Such ASC-derived enteroids and colonoids contain differentiated epithelium and mimic crypt–villus structures observed *in vivo*. While ASC organoids lack the mesenchymal and stromal components present in iPSC-derived systems, increasing access to biobanks containing annotated donor tissues is helping to overcome challenges associated with primary sample acquisition [26].

Organ-on-a-Chip devices are microengineered culture platforms that integrate living cells with microfluidic channels to recreate tissue- and organ-level physiology *in vitro* [28]. These systems emulate the dynamic mechanical and biochemical cues present *in vivo*, including shear stress, perfusion, and ECM interactions [29, 30]. Originally developed to improve predictive accuracy in drug screening pipelines, OoC systems are increasingly employed in disease modeling and mechanistic studies, particularly when traditional models fail to capture human pathophysiology [31, 32].

With advancements in stem cell and organoid biology, OoC technologies now support integrated co-cultures that replicate complex tissue interactions at micro-scale resolution [33]. Compared to spheroids and organoids, OoC devices offer superior control over the cell microenvironment and allow real-time monitoring of cellular responses under dynamic flow conditions. While they are less architecturally complex than whole tissues, they provide high physiological relevance and are particularly valuable for translational research [34, 35].

## 3D Models in EHEC and Shiga Toxins Research

The application of 3D culture systems to study bacterial pathogenesis has expanded significantly since the early 2000s. The first reported use of such systems in host–pathogen interaction studies was a 2001 investigation examining the interaction between *Salmonella enterica* serovar Typhimurium and human intestinal tissue aggregates [36, 37]. Since then, organoid-based models have been increasingly employed to explore gastrointestinal infections, particularly involving pathogens such as *Helicobacter pylori* and *Salmonella enterica* [38]. These foundational studies have provided a conceptual framework that has since been extended to research on Enterohemorrhagic *E. coli*.

### Spheroids

In the context of EHEC and its key virulence factor, Shiga toxin, 3D spheroid systems have offered critical insights into toxin-induced cellular injury and a screening platform. One of the earliest applications involved 3D human renal proximal tubular epithelial spheroids (SP-HRPTEpiS) and 3D human mini kidney spheroid model (SP3D HMKS) that were made by scaffold base to investigate Shiga toxin–induced cytotoxicity and inflammation. In this system, the regulatory mechanisms of O-GlcNAcylation in human cells exposed to Shiga toxins were first characterized in a 2D system and subsequently validated in a 3D spheroid model. This approach confirmed Stx2a-mediated injury, including secretion of inflammatory cytokines and chemokines such as IL-8 and CCL2 [39]. The enhanced spatial organization of 3D spheroids allows more physiologically relevant evaluation of Stx2a-regulated pathways, particularly in cell signaling and secretory activity.

In addition, To, Celina Z *et al*. [40] proposed that 3D spheroids could serve as a sensitive and rapid detection platform for identifying Shiga toxin–secreting *E. coli* in food samples. Conventionally, 2D cell culture assays employing African green monkey kidney (Vero) cells have been used for STEC screening and detection [41]. Importantly, the use of spheroids shortened the assay time for STEC detection from 12–16 h in 2D culture to only 6 h. 3D spheroids were created using rat-tail collagen type 1 matrix and tested with contaminated ground beef samples, with cytotoxicity assessed by measuring lactate dehydrogenase release. These results underscore the advantages of 3D spheroids, which preserve cell polarity and thereby enhance accessibility to STEC and its toxins.

Despite their physiological relevance and advantages as a culture platform, spheroids have notable limitations. Structural heterogeneity in size and morphology can create variability in experimental outcomes and affect cellular responses [22]. For example, as described by Small and Weiss [42], intestinal enteroid-derived spheroids are challenging to use in applications requiring planar monolayer formation, such as modeling the intestinal surface. Furthermore, nutrient and oxygen gradients within larger spheroids often result in hypoxia or necrosis at the core, producing conditions that do not fully reflect *in vivo* physiology. Although recent advances have improved spheroid reproducibility and biological relevance, they still fall short of replicating the complexity of native tissue microenvironments. Consequently, most spheroid-based studies of EHEC have been limited to demonstrating biological relevance after initial 2D culture or to serving as simplified detection platforms.

### Organoids

Organoid-based studies have also significantly advanced our understanding of Shiga toxin biology and EHEC pathogenesis. Human intestinal organoids (HIOs), which are derived via directed differentiation of pluripotent stem cells, recapitulate the architecture and cell-type diversity of the small intestine, one of the primary sites for EHEC colonization and toxin activity. These HIOs contain multiple differentiated epithelial cell types, including enterocytes, Paneth cells, goblet cells, and enteroendocrine cells, and express key structural markers such as villin [6]. Due to their physiological similarity to native tissue, HIOs replicate the microenvironment of the human small and large intestine, enabling precise analyses of pathogen infection and toxin activity that conventional cell lines or animal models cannot achieve.

Recent studies have demonstrated that tissue complexity influences how organoid systems respond to Shiga toxins. HIO recapitulates structural features of the distal human small intestine [43], a preferred initial attachment site for EHEC, notably *E. coli* O157:H7. Infection studies comparing nonpathogenic *E. coli* and EHEC in HIOs demonstrated that nonpathogenic *E. coli* proliferated exclusively within the lumen without causing epithelial damage, whereas EHEC infection induced progressive barrier disruption, manifested as F-actin damage at 1 h, E-cadherin loss and barrier compromise at 4 h, and complete luminal border destruction with bacterial dissemination throughout the tissue by 18 h [44]. Moreover, in HIO co-culture with human neutrophils, commensal *E. coli* remained confined to the lumen, contained by intestinal defense mechanisms such as mucus, and caused no detectable injury. In contrast, EHEC triggered severe epithelial damage, including activation of innate immune defenses, reactive oxygen species (ROS) generation, IL-8–mediated neutrophil recruitment, and multiple inflammatory responses. These findings indicate that, unlike nonpathogenic strains, EHEC causes intestinal tissue injury through virulence factor–mediated barrier disruption and immune modulation [44]. Furthermore, in HIO models incorporating both epithelial and mesenchymal components, exposure to purified Stx1a or Stx2a induced apoptosis and necrosis in both cell types [45]. This was accompanied by loss of barrier integrity and apical-to-basolateral toxin translocation. Importantly, mesenchymal injury promoted epithelial dysfunction, highlighting mesenchymal–epithelial crosstalk as a mechanism of secondary damage. Stx exposure also increased E-cadherin expression in mesenchymal cells, suggestive of an early mesenchymal-to-epithelial transition (MET)-like process. Together, these models provide a physiologically relevant platform to study EHEC adhesion, cytotoxicity, and immune responses, and to identify potential therapeutic targets [42].

Nevertheless, a major limitation of 3D organoid cultures is their incomplete cellular composition. While they contain epithelial and some mesenchymal cells, they lack vascular, neural, and immune components, preventing them from fully reproducing the complexity of native intestinal tissue [44, 45]. This shortcoming restricts studies of inflammation and immune responses [46] and limits their ability to model systemic phenomena such as metabolism or therapeutic pharmacodynamics. Furthermore, systemic complications of EHEC infection, including HUS, require translocation across the intestinal barrier into the circulation. However, the absence of a fully developed basolateral compartment in organoid cultures makes it difficult to track these metastatic pathways accurately [6]. Moreover, sourcing primary human tissue for ASC-derived organoids is not always feasible, although emerging biobanking infrastructure is helping to address this issue [47]. Although the emergence of organoid biobanks is beginning to mitigate this barrier, variability in tissue quality, donor demographics, and disease specificity remains a concern. Additionally, organoid culture systems suffer from scalability and reproducibility issues, which limit their utility in high-throughput applications or comparative studies.

### Organ-on-a-Chip

OoC platforms have further expanded the capacity to model EHEC infections in a controlled, perfused microenvironment and have evolved to replicate tissue interfaces within a single organ while mimicking increasingly complex physical and biochemical conditions [48]. A representative study by Kasendra, Magdalena *et al*. [49] used a two-channel OoC system to model human colonic epithelium infected by EHEC. This chip incorporated an interface between primary human intestinal epithelial cells and human intestinal microvascular endothelial cells (HIMECs), cultured on opposite sides of an extracellular matrix–coated membrane. Intestinal epithelial cells were grown in the upper channel to establish a luminal compartment, while HIMECs were cultured in the lower channel to represent the vascular interface. This model enabled investigation of the influence of species-specific intestinal microbial metabolites on host cell resistance to EHEC infection, comparing responses to human versus murine microbiome-derived metabolites. Furthermore, since murine models do not fully replicate the symptoms and environment of human EHEC infection, OoC systems represent a valuable first step toward directly investigating the cellular and molecular basis of human intestinal pathophysiology. Importantly, it also allowed analysis of pathogen–microbiota–host interactions under defined *in vitro* conditions. However, the study was limited by the use of microbial metabolites rather than live microorganisms, which precluded direct assessment of microbe–host cell interactions. In addition, the model did not incorporate immune cells, leaving immune contributions to EHEC pathogenesis unresolved.

While conventional systems are often limited to single-organ configurations, recent efforts have resulted in the development of multi-organ-on-chip (MOoC) platforms. These systems connect discrete tissue modules, such as gut and kidney, in a closed-loop system, thereby enabling dynamic simulation of inter-organ communication. A particularly notable study by Lee, Yugyeong *et al*. [50] introduced an integrated gut–kidney axis (GKA) chip, co-culturing Caco-2 intestinal epithelial cells and HKC-8 renal tubular epithelial cells. This platform allowed researchers to examine how Stx and antibiotic treatment affected renal injury following STEC infection. Findings from the GKA chip closely mirrored clinical observations in patients with O157:H7 infections, including toxin-mediated renal epithelial injury. Importantly, this study demonstrated that antibiotics may exacerbate renal damage in the presence of Stx, aligning with concerns over antibiotic use in STEC infection. Although our model lacked vascular and immune elements, recent studies by Maurer, Michelle *et al*. [51] and Cherne, Michelle D *et al*.[52] demonstrated that organ-on-a-chip systems incorporating immune components can effectively capture host–pathogen dynamics and hold promise as advanced platforms for future STEC research.[Fig F3]

Like this, platforms provide high resolution and physiological relevance but remain limited by technical complexity. Reproducibility can vary across laboratories and users, and device fabrication typically requires specialized expertise and equipment [40, 48]. Integrating multiple cell types into a stable, functional configuration also remains difficult, as does establishing consensus protocols for chip construction and operation [49]. Operator-dependent variability further compounds these challenges, with small differences in assembly or handling leading to inconsistent results. Moreover, the absence of standardized quality-control metrics and regulatory guidelines currently restricts the wider application of OoC devices in preclinical testing and validation. Taken together, these findings underscore the growing value of 3D model systems for dissecting EHEC pathogenesis and Stx-mediated tissue injury. Further refinements, particularly involving the integration of immune, stromal, and vascular components, will be critical for enhancing their utility as translational research tools.[Table T1]

## Conclusion

The use of 3D cell culture technologies has fundamentally reshaped how researchers study host–pathogen interactions, including those driven by enterohemorrhagic *E. coli* and its Shiga toxins. While 2D cell cultures and animal models have historically contributed valuable mechanistic insights, they fall short in capturing the full spectrum of human-specific cellular responses, particularly those involving complex tissue crosstalk and microenvironmental signaling. In contrast, 3D platforms, including spheroids, organoids, and organ-on-a-chip systems, offer improved physiological relevance and have emerged as powerful tools for modeling EHEC infection and Stx pathogenesis. These systems enable the reconstruction of key features such as epithelial barrier function, inflammatory signaling, and inter-organ dynamics, thus advancing both mechanistic understanding and preclinical evaluation of therapeutic interventions. Although current models still face limitations, especially in terms of standardization, scalability, and immune system integration, their ongoing refinement is likely to position them at the forefront of infectious disease modeling and translational microbiology.

## Figures and Tables

**Fig. 1 F1:**
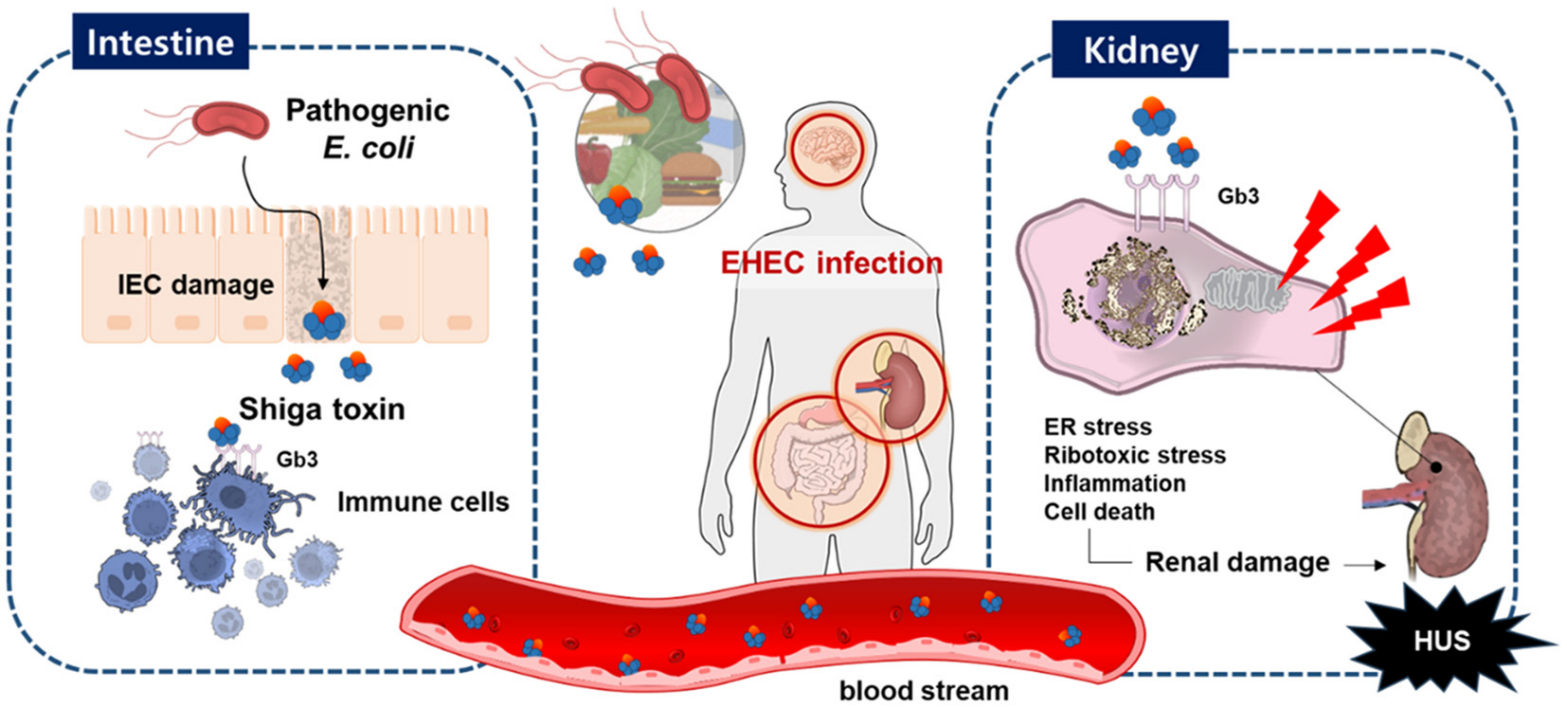
Visual summary of EHEC infection and Stxs pathogenesis. Ingestion of food contaminated with EHEC results in intestinal epithelial cell damage and the release of Stxs. The toxins activate innate immune cells, including neutrophils and monocytes, which facilitate toxin transport into the bloodstream. Once in circulation, Stxs reach glomerular endothelial cells and renal tubular epithelial cells, both of which express high levels of the Gb3 receptor. Stxs can also translocate across the intestinal mucosa into the bloodstream, ultimately disseminating to target organs such as the kidneys and central nervous system. Following Gb3-mediated endocytosis via membrane invagination, Stxs are retrogradely trafficked to the Golgi apparatus and endoplasmic reticulum. Within host cells, Stxs act as multifunctional bacterial proteins that trigger ER stress, ribotoxic stress, pro-inflammatory signaling, and apoptosis.

**Fig. 2 F2:**
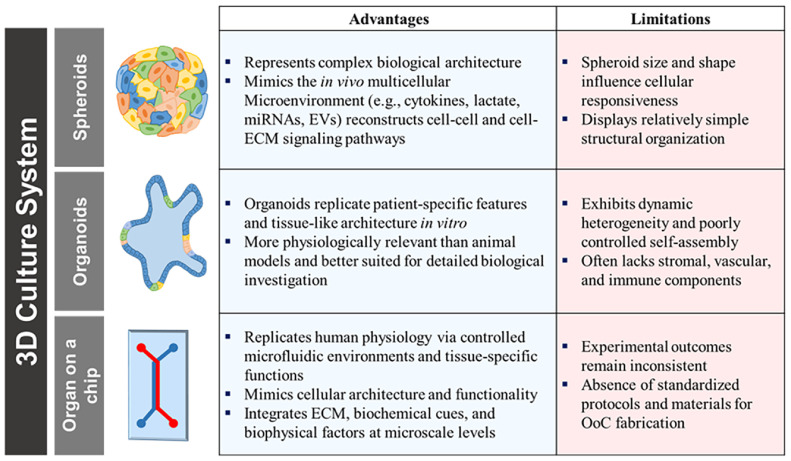
Features of 3D cell culture system. Comparison of representative 3D *in vitro* models, including spheroids, organoids, and organ-on-a-chip platforms. The schematic highlights the advantages and limitations of each system. While spheroids and organoids recapitulate aspects of *in vivo* architecture and function, organ-on-a-chip devices provide microfluidic control and greater physiological relevance.

**Fig. 3 F3:**
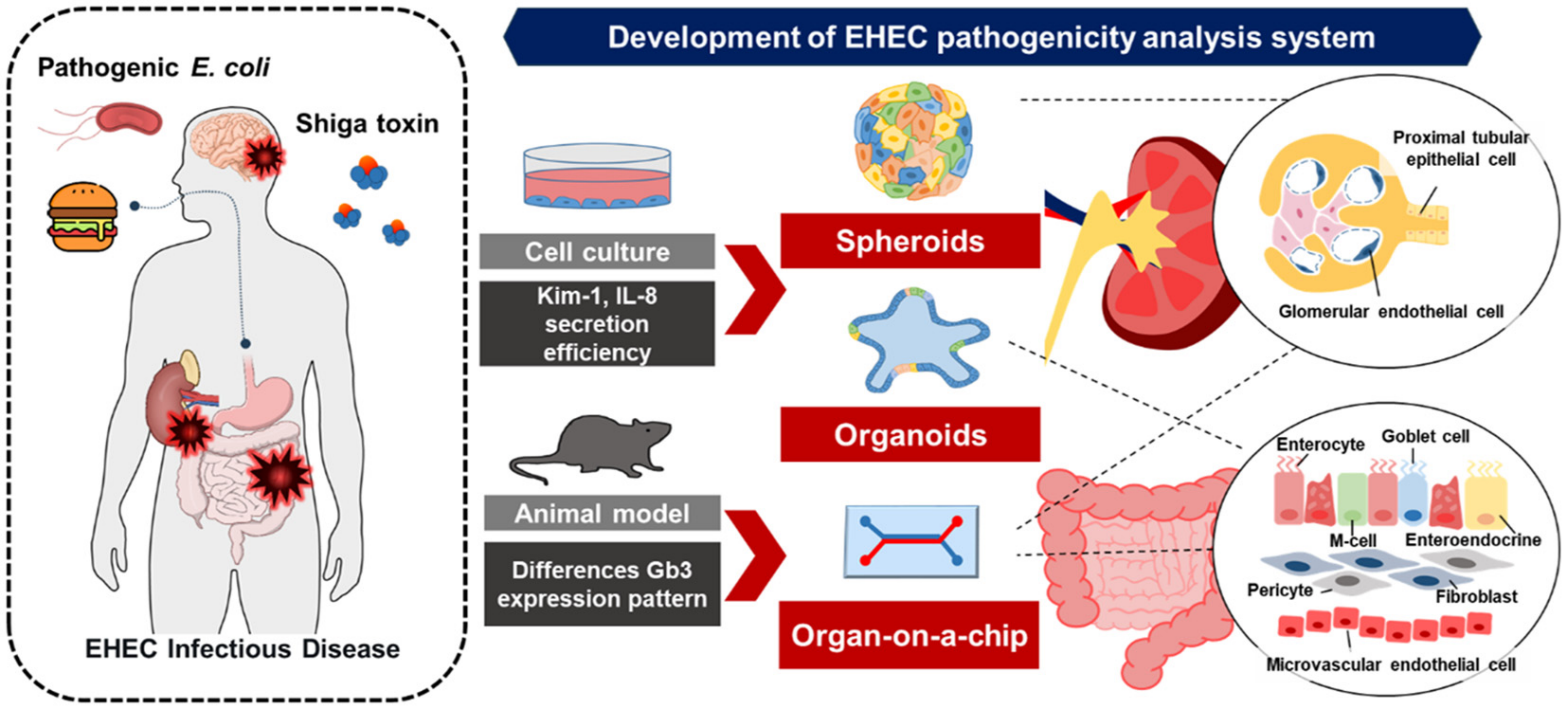
Development of an advanced model for analyzing EHECpathogenicity. Infection with enterohemorrhagic *E. coli* through contaminated food leads to the release of Shiga toxins, which cause damage to multiple organs, including the intestines, kidneys, and brain. Conventional models such as 2D cell cultures and animal systems are limited by differences in injury marker expression and toxin receptor distribution. Recent studies have demonstrated Stx-induced nephrotoxicity using renal spheroids and established more physiologically relevant infection models with intestinal organoids. Organ-on-a-chip platforms that recreate intestinal tissue interfaces or integrate gut–kidney interactions provide a further step forward in modeling EHEC pathogenesis.

**Table 1 T1:** Summary of studies and limitations of spheroids, organoids, and organ-on-a-chip platforms in EHEC infection and Shiga toxin modeling.